# Assessment of Greenhouse Gas Emissions and Carbon Sequestration in Dairy Goat Farming Systems in Northern Extremadura, Spain

**DOI:** 10.3390/ani14233501

**Published:** 2024-12-04

**Authors:** Andrés Horrillo, Paula Gaspar, Antonio Rodríguez-Ledesma, Miguel Escribano

**Affiliations:** 1 Department of Animal Production and Food Science, Faculty of Veterinary Medicine, University of Extremadura, Campus Universitario, 10003 Cáceres, Spain; andreshg@unex.es (A.H.); mescriba@unex.es (M.E.); 2 Department of Animal Production and Food Science, School of Agricultural Engineering, University of Extremadura, Avda. Adolfo Suarez, s/n, 06007 Badajoz, Spain; rledesma@unex.es

**Keywords:** carbon footprint, dairy goat farms, carbon sinks, life cycle assessment

## Abstract

Livestock farming is an activity closely linked to the environment, directly affecting its surrounding ecosystem while utilizing the natural resources it provides. Accordingly, it is essential to study these production systems in order to implement more sustainable models that allow for a balance between socioeconomic and environmental factors. The main objective of this study was to analyze the environmental impact of nine different dairy goat production systems located in northern Extremadura, Spain. The selected farms ranged from extensive and organic production models to more intensive ones. The methodology used was a life cycle assessment (LCA) to calculate the carbon footprint, following the IPCC (Intergovernmental Panel on Climate Change) guidelines. A comprehensive evaluation of emissions was conducted, considering the different global warming potentials (GWPs) established by the IPCC for the main gases associated with livestock farming. In addition, carbon sequestration (C_seq_) was incorporated into the analysis to assess the contribution of goat farming to climate change mitigation based on its production management. CH_4_ emissions were the main source (44–67%) of GHGs, followed by CO_2_ from inputs (20–48%), and finally N_2_O (7–13%). A trend was observed linking increased CH_4_ emissions to more extensive and less productive livestock systems. However, the significant role of soil as a carbon sink was highlighted, as it greatly offsets the emissions produced, demonstrating its potential mitigation due to C_seq_, particularly in more extensive models. The importance of this factor suggests that it should be taken into account in such studies to provide a realistic view of the final impact of each production system.

## Simple Summary

The objective of this study is to analyze the levels of greenhouse gas (GHG) emissions and carbon sequestration (C_seq_) in dairy goat farms in Extremadura (Spain). To achieve this, the carbon footprint of nine farms was calculated using the life cycle assessment (LCA) framework and the guidelines of the IPCC (Intergovernmental Panel on Climate Change) 2006, as well as its 2019 update, including C_seq_ calculation. The results indicate that CH_4_ is the main source of emissions, being higher in more extensive farms (3.51 kg CO_2_eq/kg fat- and protein-corrected milk (FPCM) compared to more intensive ones (1.74 kg CO_2_eq/kg FPCM). In all cases, C_seq_ significantly reduces net emissions, fully offsetting total emissions in more extensive farms by up to 100%. This facilitates greater sustainability in goat farming operations, particularly in more extensive or organic production systems, which exhibit lower levels of net emissions. The findings highlight the need to promote sustainable livestock models that implement management practices aimed at reducing GHG emissions and increasing C_seq_.

## 1. Introduction

Livestock farms are a key element for the conservation of ecosystems and for retaining rural populations due to their environmental and socioeconomic roles [1]. In the Mediterranean area, they are characterized by their extensive nature and reliance on the use of natural resources through livestock grazing. These animal production systems stand out for their diversity and complexity, with a high degree of interdependence between natural ecosystems and agricultural farms.

Globally, the total number of goats increased by 44% between 2000 and 2019, primarily in Africa and Asia. However, the trend in Europe has been declining, with a loss of 3 million heads [2]. These losses have been significant in the meat and dairy goat sectors in Spain [3]. In this context, goat farming in Spain has a long history, typically focusing on mixed meat and dairy production systems [4]. Milk production usually provides the most economic value for farmers, representing 11% of the national dairy production’s economic value in 2021, behind sheep’s milk (15%) and cow’s milk (74%) [4].

Goat production models in Spain rely on pasture-based grazing systems and are closely connected to rural regions in the southwest of the Iberian Peninsula. Goat farms are primarily located in areas unsuitable for Mediterranean agriculture [5], which is why they provide a social and environmental value that goes beyond mere economic considerations [6]. The excellent adaptability of goats, their productive versatility, recognized hardiness, and ability to graze in semi-arid areas [7] position this species as one of the most suitable for utilizing regions with challenging topography, dry climates, or low availability of natural resources, allowing them to achieve good productivity under these adverse environmental conditions [8].

The adaptability of the goat species is ideal for the development of climate change mitigation and improvement and resilience strategies in livestock farms, as proposed by the current agricultural policy (CAP) in response to the challenges posed by the current climate crisis. It is necessary to conduct studies that analyze the real impact of greenhouse gas (GHG) emissions from this species under different production models, evaluating carbon sequestration in the soil resulting from livestock grazing [9,10].

In this regard, GHG emissions from livestock activity represent one of the main environmental concerns due to their impact on global climate change [11]. Published studies show that the magnitude of these emissions varies depending on the species, the management system used for the animals [12], the methodology applied for their calculation, and the functional unit chosen to express these emissions [13].

However, despite the agro-livestock sector contributing to climate change through its emissions, it can also be part of the solution for mitigating them. This capacity is especially notable in extensive livestock production models, and it represents a key factor to consider [14]. Grazing-based livestock farming, particularly in more extensive systems, promotes the regulation of natural cycles and helps preserve soil quality, ensuring biodiversity in the surrounding environment [15]. Grazing allows for the optimal use of natural resources in pasture areas, mountain regions, or marginal ecosystems, helping to sequester carbon in the soil while enhancing its quality and biological dynamism [16]. Grazing adds organic matter to the soil, making it an activity that contributes to the conservation and protection of species and habitats that are part of each area’s biodiversity [17]. To determine the differences between more intensive production systems and those that are more extensive and/or organic, it is necessary to accurately measure the impact of grazing. This way, it can be established which system generates greater added value from both a social and environmental perspective [18].

Nevertheless, the literature on this topic is scarce, with notable works including that of Mancilla-Leytón et al. [10], which characterized the carbon footprint (CF) in four production systems of autochthonous dairy goat breeds in Andalusia (Spain), or the work of Gutiérrez-Peña et al. [9], which analyzed the CF of dairy goat systems based on their level of grazing.

In line with the context presented above, the objective of this study is to evaluate the CF of nine traditional dairy goat systems in northern Extremadura (Spain). The life cycle assessment (LCA) methodology was applied, following IPCC (Intergovernmental Panel on Climate Change) guidelines [19,20,21,22] for calculating the CF under different global warming potentials. Additionally, carbon sequestration in the soil resulting from agro-livestock activities was estimated on these farms.

## 2. Materials and Methods

### 2.1. Study Area and Data Collection

The study area is located in the southwest of the Iberian Peninsula, specifically in the Extremadura region (Spain). Extremadura is commonly known for being the region in Europe with the largest territorial area of the dehesa ecosystem [23]. However, in the northern part of the region, mountainous areas, such as the Sierra de Gata and Sierra de Gredos, stand out, reaching altitudes of over 2000 m. The climate is characterized by extreme temperatures, being cold in winter and hot in summer (with an average of 13 °C), and abundant precipitation (over 1000–1500 mm per year). Vegetation is characterized by a mosaic of dehesa, pasture, and scrubland. The predominant species are *Quercus ilex* and several species of pines (*Pinus pinaster*, *Pinus pinea*, and *Pinus sylvestris*), with a lesser presence of *Quercus pyrenaica* [24]. Given these edaphoclimatic conditions, the area has traditionally been used for extensive goat grazing. The current census of the region includes 2275 dairy goat farms, although only 372 farms were officially classified as dairy farms (Extremadura census 2022: 128,968 dairy goats) [4]. Many of these traditional farms are increasingly shifting towards more intensive production models.

Within this population, nine dairy goat farms were selected, and a case study was conducted. The case studies methodology developed by Yin [25] on his work titled “Case Study Research: Design and Methods” and it is mainly characterized by an intensive approach to an object of study or unit. In the recent literature, there are numerous studies that use the case study approach for the analysis of livestock farm management from both environmental and technical–economic approaches, for example, Bernués et al. [26], Vellenga et al. [27], Neira et al. [28], and Asai et al. [29]. Nevertheless, the study case methodology makes it impossible to extrapolate the findings statistically; however, if the cases are adequately selected, it is possible to extrapolate the findings, discover the basic principles, and contribute to scientific development [30].

These cases range from highly extensive goat farms, where animal management is closely linked to grazing and milk production is seasonal, to farms where limited grazing parcels, rest areas, and outdoor exercise yards are used permanently or semi-permanently. These more intensive farms are also characterized by maintaining stable milk production throughout the year and having high supplementary feeding requirements.

The nine dairy goat farms were monitored, with data collected on inputs, outputs, and animal management practices during 2021 and 2022. The information was obtained through fieldwork, including interviews with farmers and visits to the farms. The data obtained from each farm were recorded and digitized for subsequent analysis. All participants signed a legal consent authorizing the use of their data in this study.

### 2.2. Environmental Impact Assessment

For the environmental impact analysis, the life cycle assessment (LCA) methodology was applied [31,32]. LCA is one of the most commonly used methodologies to assess the environmental impact of a given product. The carbon footprint (CF) of dairy goat farming was calculated [12], taking into account the carbon sequestration (C_seq_) of the farms for a 100-year time horizon [33].

#### 2.2.1. Definition of System Boundaries and the Functional Unit

In the LCA, the “cradle-to-gate” approach was applied. For the system boundaries, all emissions produced both within the farm (enteric fermentation, manure management, soil management, etc.) as well as emissions resulting from the manufacturing and transportation of each of the inputs used in the analyzed production systems (feed, fuel, electricity, etc.) were considered.

The functional unit (FU) used in this LCA was fat- and protein-corrected milk (FPCM). The FU is used to compare various products and provides a common basis for comparison between different means of achieving the same outcome [34]. Therefore, in this study, emissions were expressed as kg CO_2_ equivalents per kg FPCM, as recommended by the most common LCA guidelines for the dairy sector [35].

For the standardization of goat milk, the results were expressed following the proposal of Pulina et al. [36] for dairy sheep milk:

FPCM (kg, adjusted for % of fat and protein) = raw milk (kg) × [(0.085 × fat content (%) + 0.035 × protein content (%) + 0.25)] (1)

We adjusted the equation with the verified values of goat milk from the region.

#### 2.2.2. Greenhouse Gas Emission Balance Calculation

For the calculation of GHG emissions from the farms in the study, the guidelines established by the IPCC (Intergovernmental Panel on Climate Change) (IPCC 2006, update 2019) [20] were followed. Emissions were expressed in kilograms of CO_2_ equivalents (kg CO_2_ eq). The global warming potentials (GWP) over 100 years proposed by the IPCC in the Fourth Assessment Report (AR4) [37], were used for carbon dioxide (1:CO_2_) emissions, methane (25:CH_4_) emissions, and nitrous oxide (298:N_2_O) sources. Additionally, the study also considers the GWPs proposed in the Fifth Assessment Report (AR5) [38], with feedback not included (1:CO_2_, 28:CH_4_ and 265:N_2_O) and with feedback included (1:CO_2_, 34:CH_4_ and 298:N_2_O), as well as the Sixth Assessment Report (AR6) (1:CO_2_, 27.2:CH_4_ and 273:N_2_O) [39].

CH_4_ emissions from enteric fermentation were estimated using the “Tier 2” level equations from the IPCC guidelines [21], based on gross energy [40] for each animal subcategory. Emission factors were calculated from gross energy intake and the estimated methane conversion factor. The CH_4_ conversion factor was calculated based on the equation provided by Jaurena et al. [41] (Equation (2)), as recommended for the calculation of nitrogen and phosphorus balance in ruminants in Spain [42,43]. CH_4_ conversion factors (Y_m_) specific to each animal subcategory were calculated from estimates of dry matter intake (Dm_intake_) and estimates of the neutral detergent fiber intake of the diet (NDF) [43]. The CH_4_ conversion factors for the subcategories were calculated using the following equation [41]:

(2) $$Y_{m}=3.5-0.243\times {Dm}_{intake}+{5.9\times 10}^{-2}\times NDF+{5.7\times 10}^{-2}\times DE$$

where:

Y_m_: methane conversion rate in %.

Dm_intake_: dry matter intake in kg/day.

NDF: neutral detergent fiber in the diet in %.

DE: digestibility of the energy in the diet in %.

Emissions from manure management (CH_4_ and N_2_O) were calculated following the IPCC “Tier 2” approach [21]. The estimation of CH_4_ emissions from manure management systems is calculated by animal category, the number of animals in each category, nitrogen excreted per animal category, and the distribution share within the management system. Specific data are from the “Spanish National GHG Inventory” [44] and the reports “Bases Zootécnicas para el cálculo del Balance alimentario de nitrógeno y de fósforo” [45]. For N_2_O emissions from dung and urine in pasture-based systems, calculations followed the IPCC guidelines [22] for dry climates, based on methodologies from previous studies [46].

CO_2_ emissions (off-farm emissions) include the manufacturing and transportation of each input used on the farm, including both forage and feed, the use of fossil fuels, and electricity, among other factors. Emission factors were adapted to the study area [44] and the origin of the raw materials used [47].

Along with GHG emissions, C_seq_ in soils was calculated for the farming systems with associated land areas. The concept of carbon sequestration refers to the changes in soil composition concerning carbon (C) levels. It was calculated based on the estimation of dry matter from pastures and crops, as well as soil and manure management. The amount of dry matter in the various land areas was estimated from published reports and previous studies [48]. Different land uses and some livestock management practices can significantly affect soil C levels. Good management practices applied to the soils of these farms can increase C sequestration (e.g., rotational grazing, permanent crops, no stubble burning, night penning).

In this research, it was decided to use the net C flow balance in the livestock–manure–grazing system for a 100-year horizon, as proposed by Petersen et al. [33], and later adapted to the characteristics of extensive systems by other authors [49].

### 2.3. Allocation

The allocation principle applied was economic [9], based on the income from the sale of milk and kids. Although milk is the main product in dairy goat farms, total emissions must also be allocated to the sale of kids, as they are a co-product with market value. In this study, economic allocation principles were applied based on the following data: the kids were sold at 1 month of age, with an average live weight (LW) of approximately 9.80 kg and an average monetary value of EUR 3.40 per kg of LW. The milk, in turn, had an average monetary value of EUR 0.87 per kg of raw milk. No other sources of income were observed within the scope of the study. The CF was expressed in kg of CO_2_-eq per kg FPCM.

## 3. Results

### 3.1. Main Characteristics of the Case Studies

In the analyzed goat herds, the largest group of animals consists of dairy goats, followed by young females intended for herd replacement, and a small number of adult males. Milk sales represent the primary source of income for these farms. The main characteristics, based on the calculation of indicators for each production system, are presented in the Table 1.

Among the farms analyzed, three were classified as extensive systems, two as organic production systems, and the remaining four as semi-intensive systems. A classification similar to the typologies proposed for the analyzed goat farms can be found in previous works, such as Martin et al. [50], Gaspar et al. [51] and Mena et al. [52]. In the extensive and organic systems, breeding animals and replacement females (over 6 months old) spend an average of 40% of their time grazing, following the common practice in dairy goat farming. Farmers typically milk the herd once a day, early in the morning, and after milking, the animals graze until sunset. In the more intensive systems, goats are usually milked twice a day, once in the morning and again in the afternoon, which significantly reduces their grazing time. In these systems, goats are often housed in rest enclosures with free access to pasture.

Reproductive performance ranged from 0.50 to 1.24 kids sold per goat per year, depending significantly on the production system. The annual replacement rate in dairy farms is approximately 20%. On all farms, supplementary feed was provided to lactating animals, particularly during their production phase.

### 3.2. Results of the GHG Emissions Balance by Farm

**Figure 1:**
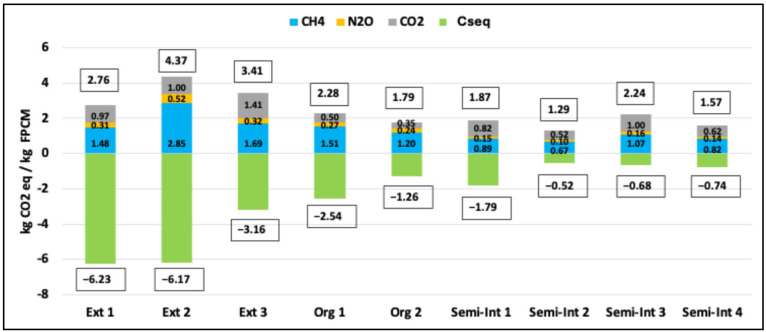
Greenhouse gas (GHG) emissions balance of the farms in the study in kg CO_2_ eq/kg FPCM and GWP proposed in AR4. (Ext: extensive system, Org: organic system; Semi-Int: semi-intensive system; C_seq_: carbon sequestration).

The results for CF emissions and C_seq_ for the farms in the study, using a 100-year time horizon and GWP (AR4) values of 1 for CO_2_, 25 for CH_4_, and 298 for N_2_O, are shown in Figure 1. The results for the alternative GWP values are shown in Figures S1–S4 in the Supplementary Materials.

The average total GHG emissions for the extensive farms were 3.51 ± 0.81 kg CO_2_eq/kg FPCM, for the organic farms they were 2.03 ± 0.34 kg CO_2_eq/kg FPCM, and for the semi-intensive farms they were 1.74 ± 0.41 kg CO_2_eq/kg FPCM. In general, the highest percentage of emissions was related to CH_4_ emissions, which include enteric fermentation and manure management, with an average value of 56%, followed by CO_2_ emissions at 34% and N_2_O emissions at 10%. For extensive farms, CH_4_ emissions ranged from a maximum of 2.85 to a minimum of 1.48 kg CO_2_eq/kg FPCM (65.22–53.56%, respectively, of total emissions), for organic farms from 1.51 to 1.20 kg CO_2_eq/kg FPCM (66.32–66.84%, respectively, of total emissions), and for semi-intensive farms from 1.07 to 0.67 kg CO_2_eq/kg FPCM (47.96–51.47%, respectively, of total emissions).

The estimated C_seq_ in the soils of the studied farms ranged from a maximum of 6.23 kg CO_2_eq/kg FPCM to a minimum of 0.52 kg CO_2_eq/kg FPCM, showing significant variability between farms. For the extensive farms, the average was 5.19 ± 1.75 kg CO_2_eq/kg FPCM, for organic farms it was 1.90 ± 0.90 kg CO_2_eq/kg FPCM, and for the semi-intensive farms, it was 0.94 ± 0.58 kg CO_2_eq/kg FPCM. The inclusion of carbon sequestration balances emissions by around 222% in more extensive systems and by approximately 30–96% in more intensive systems. In some cases, C_seq_ completely offsets GHG emissions, as seen in the first two extensive farms (Ext 1 and Ext 2) and one of the organic farms (Org 1).

In Table 2, it can be observed how the final GHG emissions results vary depending on the assumed GWP values.

As shown in Table 2, the results vary depending on the chosen GWP, with higher GHG emission levels observed in GWP AR5b. This can be explained by the fact that, as mentioned earlier, CH_4_ emissions—both from enteric fermentation and manure management—are the largest contributors to the total emissions in the goat farms studied. In the GWP of AR5b, CH_4_ is assigned the highest conversion factor (34:CH_4_).

**Figure 2:**
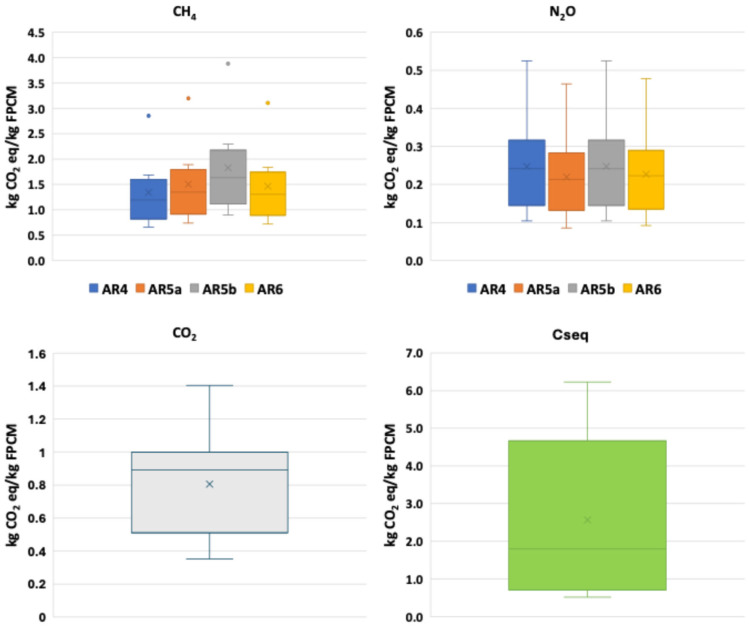
GHG emissions results for a 100-year horizon and the different GWPs (AR4: Fourth Assessment Report; AR5a: Fifth Assessment Report with feedback not included; AR5b: Fifth Assessment Report with feedback included; AR6: Sixth Assessment Report; C_seq_: carbon sequestration).

This can be more clearly seen in Figure 2, which details the results by type of GHG according to the selected GWP values and for a 100-year time horizon.

Figure 2 shows the differences in the level of contribution of CH_4_ and N_2_O emissions in the farms according to the chosen GWP. It can be observed that they are higher for CH_4_ in the GWP case of AR5b (34:CH_4_) and lower for N_2_O in AR4 and AR5b (298:N_2_O).

## 4. Discussion

### 4.1. Intensification vs. Extensification and Greenhouse Gas (GHG) Emission Levels

The increasing concern over climate change and the significant contribution of food production to greenhouse gas (GHG) emissions underscore the need for new production models that can help offset or mitigate these emissions [53]. In our analysis of emissions per kilogram of milk produced, it was found that extensive and/or organic goat farms generate higher emission levels compared to intensive farms, mainly due to the lower milk productivity of their herds [9]. Conversely, Steinfeld et al. [54] and Sintori et al. [55], highlight the significant role that livestock intensification and feed production play as key contributors to global GHG emissions.

Consistent with the comments above, when we analyze farms solely based on their productivity levels (kg produced), it aligns with Recktenwald and Ehrhardt’s [56] findings that emission levels are lower on more intensively managed farms, primarily due to these farms producing less methane (CH_4_) per unit of product.

The previous discussion may seem contradictory, but it serves to open the current debate on how to measure greenhouse gas emissions—whether at a local or global level, and also at what scale or spatial reference level.

In any case, the choice of a functional unit (FU) for farming and production systems must be appropriate. The exclusive use of a FU based on final products (kg of milk or meat) has been predominant in published studies on the carbon footprint (CF) [12]. However, in extensive systems, a functional unit based on grazing area, in addition to one based on products, can provide more information about their sustainable management [57], leading to completely different results.

But it is not only the chosen FU that can yield different results. It is also observed that the application of different global warming potentials (GWPs) generates varying outcomes, as shown in the work of Reyes-Palomo et al. [58].

In the near future, the carbon market could become a key incentive for the ecological transition of sustainable livestock farming. For its development, a regulatory framework is needed to standardize methodologies for monitoring, accounting, reporting, and verifying GHG emissions and carbon sequestration in soils, as well as to establish penalties for non-compliance.

The Commission has developed a proposal to establish an EU Carbon Removal Certification Framework to ensure that economic value is placed on practices that increase carbon removal and storage, based on scientifically proven measurements. This research can provide valuable information for the development of a framework tailored to pasture-based livestock systems.

### 4.2. Impact of Carbon Sequestration in LCA

Carbon sequestration (C_seq_) involves changes in the levels of carbon permanently stored in the soil. These changes occur in agricultural soils due to the action of crop residues, livestock activity related to grazing, and the manure spread on the fields. Additionally, different land uses and certain livestock management practices can significantly affect soil carbon levels.

As a general rule, C_seq_ is not included in LCAs and is only particularly relevant in studies that analyze extensive, land-based systems [9,46]. Excluding this sequestration could lead to an overestimation of emissions from pasture-based extensive systems [59,60].

For the goat farms analyzed, C_seq_ was found to significantly offset GHG emissions, even exceeding 100% in the case of extensive and organic farms. These findings have been highlighted in other studies, which show that including C_seq_ reduces the net emissions balance in livestock operations. In the studies by Canan [61] and Reyes-Palomo et al. [58], it was observed that in agro-silvopastoral systems, C_seq_ can significantly mitigate GHG emissions.

The results show that extensive goat farms have higher C_seq_ (5.19 ± 1.75 kg CO_2_eq/kg fat- and protein-corrected milk (FPCM) compared to less extensive systems, such as semi-intensive goat farms (0.94 ± 0.58 kg CO_2_eq/kg FPCM). These findings are consistent with those of authors, like Gutiérrez-Peña et al. [9], who compared C_seq_ in goats across various systems with different levels of grazing. They are also similar to the results obtained by Escribano et al. [62] for sheep in semi-arid pastures of Extremadura (Spain), as well as those of Batalla et al. [16] for sheep farming in northern Spain.

Higher C_seq_ is strongly associated with lower stocking rates, underscoring the role of carbon sinks in mitigating climate change and significantly reducing the overall CF. Reyes-Palomo et al. [40] and Veysset et al. [63] suggest that, if carbon sequestration were considered, compensation rates could offset between 40% and 70% of total emissions in grazing-based systems. The FAO [64] estimates that agricultural and livestock soils have the potential to sequester more than 10% of CO_2_ emissions. Recent studies have demonstrated how C_seq_ can be optimized through proper livestock management practices, such as rotational grazing and appropriate grazing intensity [65].

In extensive livestock farms within dehesa ecosystems, C_seq_ primarily depends on plant residues and, consequently, on the amount of available pastureland [18]. On a larger scale, farms with more land area have greater potential to sequester carbon. It is important to clarify that C_seq_ is linked to pasture productivity, meaning the amount of dry matter produced. Pasture productivity, in turn, depends on the farm’s geographic location, grazing management practices, and the application of soil improvement techniques. In addition to the carbon contributed by plant residues, the contribution of animal manure to C_seq_ should also be considered [18].

What is described above is evident in our analysis of the systems from a territorial perspective, leading to the conclusion that extensive systems have a lower impact per unit of land area compared to more intensive models [66]. In addition, those systems have a notable capacity for carbon sequestration.

The applied grazing systems can provide ecosystem services, such as carbon sequestration in the soil, the maintenance of ecosystems and their biodiversity, soil restoration, and pasture production. It is essential to value these services and integrate them into the evaluation of emissions and the impact of a livestock farm or production system [67].

### 4.3. Effective Goat Management as a Strategy to Reduce Emissions

The environmental impacts of livestock production largely depend on the farming systems implemented within their overall production approach, which can, in turn, be influenced by the technical management practices employed. Not all animal production systems emit GHGs with the same intensity.

In this sense, Marino et al. [68] highlighted the interconnected nature of GHG emissions and their impact on climate change in small ruminant production. Similarly, Salcedo et al. [69] explored strategies to reduce emissions per hectare and per unit of product, illustrating the potential benefits of feed substitution and other management practices. Mancilla-Leytón et al. [10] monitored various farms, considering both GHG emissions and carbon sequestration, concluding that the latter can mitigate the GHGs emitted by livestock farming.

Goat farms can specifically reduce their emission levels by adopting proper management practices. In extensive farming systems, improving reproductive performance, managing grazing effectively, and enhancing forage quality can significantly reduce farm emissions. For intensive farms, optimizing manure management and using locally sourced feed can also substantially lower GHG emissions [46].

## 5. Conclusions

Studies on the carbon footprint (CF) of livestock are receiving growing attention in scientific literature, but many focus primarily on more intensive models, with few considering carbon sequestration and its mitigation potential.

This study examines the climate impact of nine dairy goat farms in Extremadura with different farming systems. When emissions are measured per kilogram of milk as the functional unit, intensive farms show lower emissions due to their higher productivity. However, incorporating carbon sequestration reveals the mitigation potential of more extensive grazing systems, highlighting their ability to offset and reduce the final GHG emissions. Specifically, in the farms analyzed, it was observed that carbon sequestration (C_seq_) is higher in more extensive systems, resulting in a lower net emissions balance.

Integrating C_seq_ into the LCA of extensive farms, along with identifying livestock practices that help farms adapt to potential climate change scenarios, can be key to advising farmers on more sustainable management models with lower environmental impacts.

The livestock sector can play a key role in mitigating climate change through the adoption of improved technologies and proper land use. However, as the study shows, the use of different methodologies, emission factors, and global warming potentials (GWPs) can produce varying results, making it difficult to compare outcomes across farms. This fact can also affect the implementation of different agricultural policies.

## Figures and Tables

**Table 1:** Characteristics of the studied farms.

Farms	Indicators	Unit	Ext 1	Ext 2	Ext 3	Org 1	Org 2	Semi-Int 1	Semi-Int 2	Semi-Int 3	Semi-Int 4
Utilised Agricultural Area	Total area	ha ^1^	551.00	600.00	127.01	220.10	137.00	290.00	158.00	103.00	225.40
Herd structure	Dairy goats	Heads	200	440	150	231	300	241	400	400	540
	Adult males	Heads	8	20	4	7	4	6	8	7	13
	Replacement females	Heads	25	102	25	31	50	31	90	100	80
	Replacement males	Heads	2	8	7	2	0	1	6	3	0
Stocking rate	Goat stoking rate	LU ^2^/ha	0.07	0.07	0.24	0.20	0.42	0.16	0.49	0.76	0.45
Feed inputs	Straw	kg/goat/year	262.50	0.00	0.00	48.48	30.00	210.37	73.75	151.00	101.85
	Hay	kg/goat/year	82.55	0	0	0	0	0	0	0	96.29
	Concentrate goat feed	kg/goat/year	75.00	0.00	97.33	102.00	105.13	300.00	300.88	250.00	293.42
	Cereal goat mix	kg/goat/year	90.00	84.34	0.00	0.00	0.00	0.00	0.00	2.00	0.00
Grazing	Grazing time	Hours	8	9.5	5	8.5	9	6	5	4	5
Milking	Milking sessions	Number/day	1	1	2	1	2	2	2	2	2
Production	Milk sold	kg/goat/year	154.65	82.95	128.53	151.30	223.38	299.46	433.02	257.75	318.85
	Kids sold	Number of kids/goat/year	1.00	0.68	1.21	0.87	0.50	1.24	0.63	0.63	0.86
	Kids’ age	Days	30	30	35	30	30	30	32	35	32
	Kids’ sale weight	kg/kid	10.5	9.5	10	11.5	8.5	10	9	10	9.50

Ext: extensive system; Org: organic system; Semi-Int: semi-intensive system; ha ^1^: hectares; LU ^2^: livestock unit.

**Table 2:** GHG emissions of the farms in the study according to GWP (kg CO_2_eq/kg FPCM).

Total GHG Emissions	Ext 1	Ext 2	Ext 3	Org 1	Org 2	Semi-Int 1	Semi-Int 2	Semi-Int 3	Semi-Int 4
AR4	2.76	4.37	3.41	2.28	1.79	1.87	1.29	2.24	1.57
AR5a	2.91	4.66	3.58	2.43	1.91	1.94	1.35	2.35	1.66
AR5b	3.29	5.40	4.02	2.82	2.23	2.16	1.53	2.63	1.87
AR6	2.87	4.58	3.54	2.39	1.88	1.93	1.33	2.32	1.64

Ext: extensive system; Org: organic system; Semi-Int: semi-intensive system; AR4: Fourth Assessment Report; AR5a: Fifth Assessment Report with feedback not included; AR5b: Fifth Assessment Report with feedback included; AR6: Sixth Assessment Report.

## Data Availability

Dataset available on request from the authors.

## Supplementary Materials

The following supporting information can be downloaded at: https://www.mdpi.com/article/10.3390/ani14233501/s1, Figure S1: GHG emissions balance of the farms in the study in kg CO_2_ eq/kg FPCM and GWP proposed in the Fifth Assessment Report [38], with feedback not included (AR5a); Figure S2: GHG emissions balance of the farms in the study in kg CO_2_ eq/kg FPCM and GWP proposed in the Fifth Assessment Report [38], with feedback included (AR5b); Figure S3: GHG emissions balance of the farms in the study in kg CO_2_ eq/kg FPCM and GWP proposed in the Sixth Assessment Report [39] (AR6); Figure S4: GHG emissions balance of the farms in the study in kg CO_2_ eq/hectares and GWP proposed in the Fourth Assessment Report [37] (AR4) (ha: hectares).

## References

[1] Bernués A., Ruiz R., Olaizola A., Villalba D., Casasús I. (2011). Sustainability of Pasture-Based Livestock Farming Systems in the European Mediterranean Context: Synergies and Trade-Offs. Livest. Sci..

[2] MAPA (2020). Informe Sectorial Ovino y Caprino 2020. Subdirección General de Producciones Ganaderas y Cinegéticas. Dirección General de Producciones y Mercados Agrarios.

[3] MAPA (2024). Caraterización Del Sector Ovino y Caprino En España. Subdirección General de Producciones Ganaderas y Cinegéticas, Dirección General de Producciones y Mercados Agrarios.

[4] MAPA (2023). El Sector Ovino y Caprino de Leche En Cifras: Principales Indicadores Económicos. Subdirección General de Producciones Ganaderas y Cinegéticas. Dirección General de Producciones y Mercados Agrarios.

[5] Castel J.M., Ruiz F.A., Mena Y., Sánchez-Rodríguez M. (2010). Present Situation and Future Perspectives for Goat Production Systems in Spain. Small Rumin. Res..

[6] Castel J.M., Mena Y., Ruiz F.A., Gutiérrez R. (2012). Situación y Evolución de Los Sistemas de Producción Caprina En España. Tierras. Caprino.

[7] Belanche A., Martín-Collado D., Rose G., Yáñez-Ruiz D.R. (2021). A Multi-Stakeholder Participatory Study Identifies the Priorities for the Sustainability of the Small Ruminants Farming Sector in Europe. Animal.

[8] Daza A., Fernández C., Sánchez A. (2004). Sistemas de Produccion. Ganado Caprino: Producción, Alimentación y Sanidad.

[9] Gutiérrez-Peña R., Mena Y., Batalla I., Mancilla-Leytón J.M. (2019). Carbon Footprint of Dairy Goat Production Systems: A Comparison of Three Contrasting Grazing Levels in the Sierra de Grazalema Natural Park (Southern Spain). J. Environ. Manag..

[10] Mancilla-Leytón J.M., Morales-Jerrett E., Muñoz-Vallés S., Mena Y. (2023). A Comparative Analysis of Carbon Footprint in the Andalusian Autochthonous Dairy Goat Production Systems. Animals.

[11] FAO (2017). Livestock Solutions for Climate Change.

[12] Buratti C., Fantozzi F., Barbanera M., Lascaro E., Chiorri M., Cecchini L. (2017). Carbon Footprint of Conventional and Organic Beef Production Systems: An Italian Case Study. Sci. Total Environ..

[13] de Vries M., van Middelaar C.E., de Boer I.J.M. (2015). Comparing Environmental Impacts of Beef Production Systems: A Review of Life Cycle Assessments. Livest. Sci..

[14] Herrera P.M. (2020). Ganadería y Cambio Climático: Un Acercamiento En Profundidad.

[15] Ruiz Mirazo J. (2011). Las Áreas Pasto-Cortafuegos: Un Sistema Silvopastoral Para La Prevención de Incendios Forestales.

[16] Batalla I., Knudsen M.T., Mogensen L., Del Hierro Ó., Pinto M., Hermansen J.E. (2015). Carbon Footprint of Milk from Sheep Farming Systems in Northern Spain Including Soil Carbon Sequestration in Grasslands. J. Clean. Prod..

[17] Zabalza S., Peiteado C., Carricondo A., Astrain C., Toom Md V.M. (2017). Sistemas de Alto Valor Natural: Análisis de La Programación de Desarrollo Rural 2014–2020. Medidas Agroambiente y Clima.

[18] Eldesouky A., Mesias F.J., Elghannam A., Escribano M. (2018). Can Extensification Compensate Livestock Greenhouse Gas Emissions? A Study of the Carbon Footprint in Spanish Agroforestry Systems. J. Clean. Prod..

[19] Eggleston H.S., Buendia L., Miwa K., Ngara T., Tanabe K., IPCC (2006). IPCC Guidelines for National Greenhouse Gas Inventories. Prepared by the National Greenhouse Gas Inventories Programme.

[20] Calvo Buendia E., Tanabe K., Kranjc A., Baasansuren J., Fukuda M., Ngarize S., Osako A., Pyrozhenko Y., Shermanau P., Federici S., IPCC (2019). Refinement to the 2006 IPCC Guidlines for National Greenhouse Gas Inventories.

[21] Gavrilova O., Leip A., Dong H., MacDonald J.D., Bravo C.A.G., Amon B., Rosales R.B., del Prado A., de Lima M.A., Widiawati Y. (2019). Volume 4: Agriculture, Forestry and Other Land Use. Chapter 10: Emissions Form Livestock and Manure Management. 2019 Refinement to the 2006 IPCC Guidelines for National Greenhouse Gas Inventories.

[22] Hergoualc’h K., Akiyama H., Bernoux M., Chirinda N., del Prado A., Kasimir A., McDonald J.D., Ogle S., Regina K., van der Weerden T.J. (2019). Volume 4: Agriculture, Forestry and Other Land Use. Chapter 11: N_2_O Emissions from Managed Soils, and CO_2_ Emissions from Lime and Urea Application. 2019 Refinement to the 2006 IPCC Guidelines for National Greenhouse Gas Inventories.

[23] den Herder M., Moreno G., Mosquera-Losada R.M., Palma J.H.N., Sidiropoulou A., Santiago Freijanes J.J., Crous-Duran J., Paulo J.A., Tomé M., Pantera A. (2017). Current Extent and Stratification of Agroforestry in the European Union. Agric. Ecosyst. Environ..

[24] Moreno González A., Ruiz I.M., Luis Del Pozo Barrón J., María J., Pérez G., De Vega Fernández I., Rodríguez J., Fernando G., Martín H., García A. (2022). Informe Ambiental De Extremadura 2022 Publica.

[25] Yin R.K. (1984). Case Study Research and Applications: Design and Methods.

[26] Bernués A., Rodríguez-Ortega T., Olaizola A.M., Ripoll Bosch R. (2017). Evaluating Ecosystem Services and Disservices of Livestock Agroecosystems for Targeted Policy Design and Management. Grassl. Sci. Eur..

[27] Vellenga L., Qualitz G., Drastig K. (2018). Farm Water Productivity in Conventional and Organic Farming: Case Studies of Cow-Calf Farming Systems in North Germany. Water.

[28] Neira D.P., Montiel M.S., Fernández X.S. (2014). Energy Indicators for Organic Livestock Production: A Case Study from Andalusia, Southern Spain. Agroecol. Sustain. Food Syst..

[29] Asai M., Moraine M., Ryschawy J., de Wit J., Hoshide A.K., Martin G. (2018). Critical Factors for Crop-Livestock Integration beyond the Farm Level: A Cross-Analysis of Worldwide Case Studies. Land Use Policy.

[30] Flyvbjerg B. (2006). Five Misunderstandings About Case-Study Research. Qual. Inq..

[31] (2006). Environmental Management—Life Cycle Assessement—Requirements and Guidelines.

[32] (2006). Environmental Management—Life Cycle Assessement—Requirements and Guidelines.

[33] Petersen B.M., Knudsen M.T., Hermansen J.E., Halberg N. (2013). An Approach to Include Soil Carbon Changes in Life Cycle Assessments. J. Clean. Prod..

[34] Owsianiak M., Laurent A., Bjørn A., Hauschild M.Z. (2014). IMPACT 2002+, ReCiPe 2008 and ILCD’s Recommended Practice for Characterization Modelling in Life Cycle Impact Assessment: A Case Study-Based Comparison. Int. J. Life Cycle Assess..

[35] Vagnoni E., Franca A. (2018). Transition among Different Production Systems in a Sardinian Dairy Sheep Farm: Environmental Implications. Small Rumin. Res..

[36] Pulina G., Macciotta N., Nudda A. (2005). Milk Composition and Feeding in the Italian Dairy Sheep. Ital. J. Anim. Sci..

[37] Metz B., Davidson O.R., Bosch P.R., Dave R., Meyer L.A., IPCC (2007). Climate Change 2007: Mitigation. Contribution of Working Group III to the Fourth Assessment Report of the Inter-Governmental Panel on Climate Change.

[38] Edenhofer O., Pichs-Madruga R., Sokona Y., Farahani E., Kadner S., Seyboth K., Adler A., IPCC (2014). Climate Change 2014: Mitigation of Climate Change. Contribution of Working Group III to the Fifth Assessment Report of the Intergovernmental Panel on Climate Change.

[39] Shukla P.R., Skea J., Slade R., Al Khourdajie A., van Diemen R., McCollum D., Pathak M., Some S., Vyas P., Fradera R., IPCC (2023). Intergovernmental Panel on Climate Change (2022).

[40] Reyes-Palomo C., Aguilera E., Llorente M., Díaz-Gaona C., Moreno G., Rodríguez-Estévez V. (2022). Carbon Sequestration Offsets a Large Share of GHG Emissions in Dehesa Cattle Production. J. Clean. Prod..

[41] Jaurena G., Cantet J.M., Arroquy J.I., Palladino R.A., Wawrzkiewicz M., Colombatto D. (2015). Prediction of the Ym Factor for Livestock from On-Farm Accessible Data. Livest. Sci..

[42] MAPA (2019). Ovino. Bases Zootécnicas Para El Cálculo Del Balance Alimentario de Nitrógeno y de Fósforo.

[43] MAPA (2021). Caprino. Bases Zootécnicas Para El Cálculo Del Balance Alimentario de Nitrógeno y de Fósforo.

[44] MITECO (2024). Informe de Inventario Nacional de Emisiones de Gases de Efecto Invernadero.

[45] MAPA (2021). Balance Del Nitrógeno En La Agricultura Española (Años 1990–2019).

[46] Escribano M., Horrillo A., Mesías F.J. (2022). Greenhouse Gas Emissions and Carbon Sequestration in Organic Dehesa Livestock Farms. Does Technical-Economic Management Matters?. J. Clean. Prod..

[47] Bochu J.-L., Metayer N., Bordet C., Gimaret M. (2013). Development of Carbon Calculator to Promote Low Carbon Farming Practices—Methodological Guidelines (Methods and Formula). Deliverable to EC-JRC-IES by Solagro.

[48] Olea L., San Miguel-Ayanz A. The Spanish Dehesa: A Traditional Mediterranean Silvopastoral System Linking Production and Nature Conservation. Proceedings of the Sustainable Grassland Productivity: 21st General Meeting of the European Grassland Federation.

[49] Horrillo A., Gaspar P., Escribano M. (2020). Organic Farming as a Strategy to Reduce Carbon Footprint in Dehesa Agroecosystems: A Case Study Comparing Different Livestock Products. Animals.

[50] Martín M., Escribano Sánchez M., Mesías Díaz M., Rodriguez de Ledesma A., Pulido García F. (2001). Sistemas Extensivos de Producción Animal. Arch. Zootec..

[51] Gaspar P., Escribano A.J., Mesías F.J., Escribano M., Pulido A.F. (2011). Goat Systems of Villuercas-Ibores Area in SW Spain: Problems and Perspectives of Traditional Farming Systems. Small Rumin. Res..

[52] Mena Y., Ruiz-Mirazo J., Ruiz F.A., Castel J.M. (2016). Characterization and Typification of Small Ruminant Farms Providing Fuelbreak Grazing Services for Wildfire Prevention in Andalusia (Spain). Sci. Total Environ..

[53] Herrero M., Havlík P., Valin H., Notenbaert A., Rufino M.C., Thornton P.K., Blümmel M., Weiss F., Grace D., Obersteiner M. (2013). Biomass Use, Production, Feed Efficiencies, and Greenhouse Gas Emissions from Global Livestock Systems. Proc. Natl. Acad. Sci. USA.

[54] Steinfeld H., Gerber P., Wassenaar T., Castel V., Rosales M., de Haan C. (2006). Livestock’s Long Shadow: Environmental Issues and Options.

[55] Sintori A., Tsiboukas K., Zervas G. (2013). Evaluating Socio-Economic and Environmental Sustainability of the Sheep Farming Activity in Greece: A Whole-Farm Mathematical Programming Approach. Methods and Procedures for Building Sustainable Farming Systems.

[56] Recktenwald E.B., Ehrhardt R.A. (2024). Greenhouse Gas Emissions from a Diversity of Sheep Production Systems in the United States. Agric. Syst..

[57] Röös E., Sundberg C., Tidåker P., Strid I., Hansson P.-A. (2013). Can Carbon Footprint Serve as an Indicator of the Environmental Impact of Meat Production?. Ecol. Indic..

[58] Reyes-Palomo C., Aguilera E., Llorente M., Díaz-Gaona C., Moreno G., Rodríguez-Estévez V. (2023). Free-Range Acorn Feeding Results in Negative Carbon Footprint of Iberian Pig Production in the Dehesa Agro-Forestry System. J. Clean. Prod..

[59] Knudsen M.T., Dorca-Preda T., Djomo S.N., Peña N., Padel S., Smith L.G., Zollitsch W., Hörtenhuber S., Hermansen J.E. (2019). The Importance of Including Soil Carbon Changes, Ecotoxicity and Biodiversity Impacts in Environmental Life Cycle Assessments of Organic and Conventional Milk in Western Europe. J. Clean. Prod..

[60] Reyes-Palomo C., Díaz-Gaona C., Sanz-Fernández S., Muñoz-Cobos I., Aguilera E., Rodríguez-Estévez V. (2024). Carbon Footprint of an Extensively Raised, Low-Productivity Sheep Population. Agriculture.

[61] Canan S. (2023). Reducing Carbon Emission in the Goat Farms by Switching from Conventional Goat Farming to Green Goat Farm Typology. Front. Environ. Sci..

[62] Escribano M., Elghannam A., Mesias F.J. (2020). Dairy Sheep Farms in Semi-Arid Rangelands: A Carbon Footprint Dilemma between Intensification and Land-Based Grazing. Land Use Policy.

[63] Veysset P., Lherm M., Bébin D. (2010). Energy Consumption, Greenhouse Gas Emissions and Economic Performance Assessments in French Charolais Suckler Cattle Farms: Model-Based Analysis and Forecasts. Agric. Syst..

[64] FAO (2017). Voluntary Guidelines for Sustainable Soil Management Food and Agriculture Organization of the United Nations Rome, Italy.

[65] McSherry M.E., Ritchie M.E. (2013). Effects of Grazing on Grassland Soil Carbon: A Global Review. Glob. Change Biol..

[66] Stanley P.L., Rowntree J.E., Beede D.K., DeLonge M.S., Hamm M.W. (2018). Impacts of Soil Carbon Sequestration on Life Cycle Greenhouse Gas Emissions in Midwestern USA Beef Finishing Systems. Agric. Syst..

[67] Ripoll-Bosch R., de Boer I.J.M., Bernués A., Vellinga T.V. (2013). Accounting for Multi-Functionality of Sheep Farming in the Carbon Footprint of Lamb: A Comparison of Three Contrasting Mediterranean Systems. Agric. Syst..

[68] Marino R., Atzori A.S., D’Andrea M., Iovane G., Trabalza-Marinucci M., Rinaldi L. (2016). Climate Change: Production Performance, Health Issues, Greenhouse Gas Emissions and Mitigation Strategies in Sheep and Goat Farming. Small Rumin. Res..

[69] Salcedo G., García O., Jiménez L., Gallego R., González-Cano R., Arias R. (2022). GHG Emissions from Dairy Small Ruminants in Castilla-La Mancha (Spain), Using the ManleCO_2_ Simulation Model. Animals.

