# Supplementary material for: Assessment of Greenhouse Gas Emissions and Carbon Sequestration in Dairy Goat Farming Systems in Northern Extremadura, Spain
Source: Animals (Basel). 2024 Dec 4;14(23):3501. doi: 10.3390/ani14233501 (PMC11640683; doi:10.3390/ani14233501)
Supplement: Supplementary file 1 [file animals-14-03501-s001.zip › animals-3274303-supplementary.pdf]

Supplementary Materials:

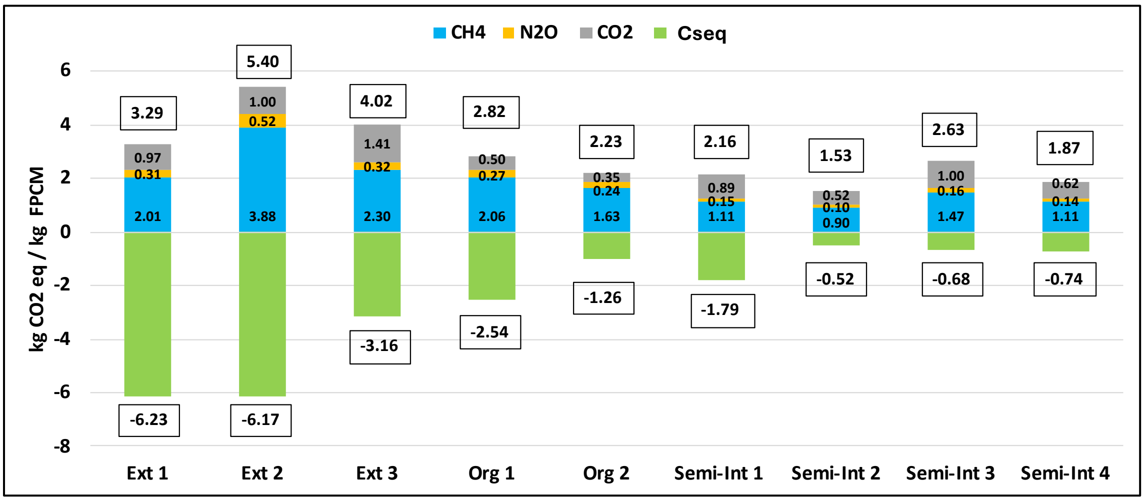

Figure S1. GHG Emissions Balance of the farms in the study in kg CO_2 eq/kg FPCM and GWP proposed in the Fifth Assessment Report [1], with Feedback Not Included (AR5a).

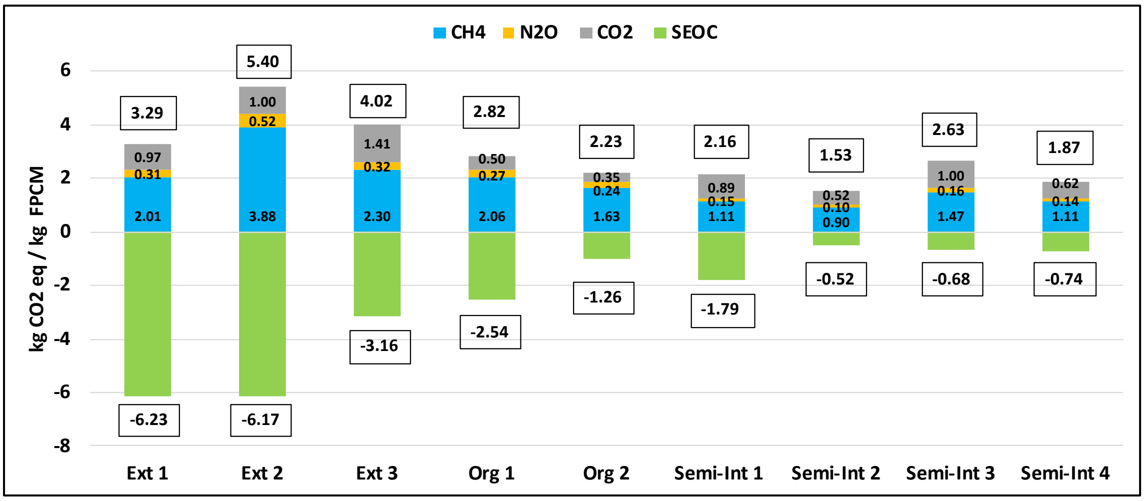

Figure S2. GHG Emissions Balance of the farms in the study in kg CO2 eq/ kg FPCM and GWP proposed in the Fifth Assessment Report [1], with Feedback Included (AR5b).

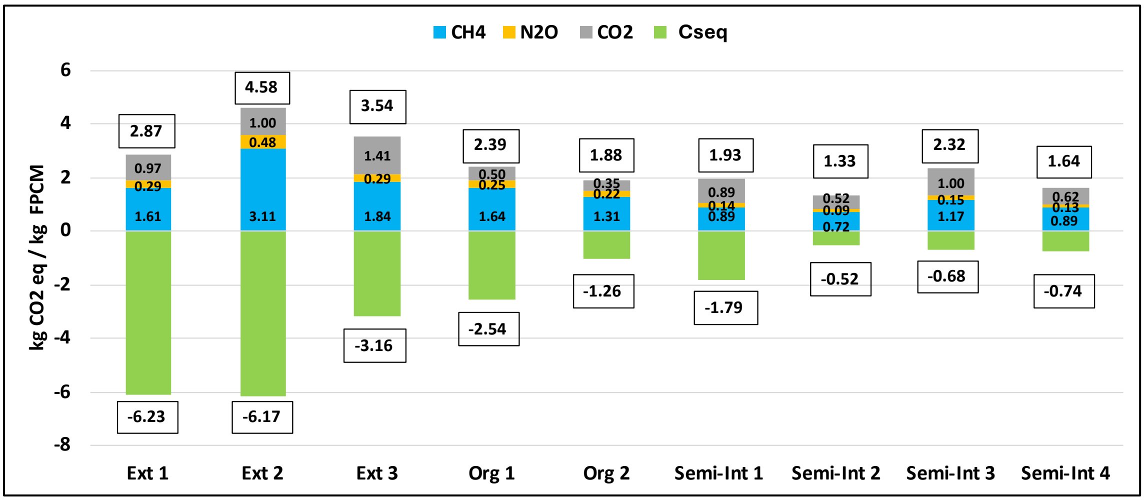

Figure S3. GHG Emissions Balance of the farms in the study in kg CO_2 eq/kg FPCM and GWP proposed in the Sixth Assessment Report [2] (AR6).

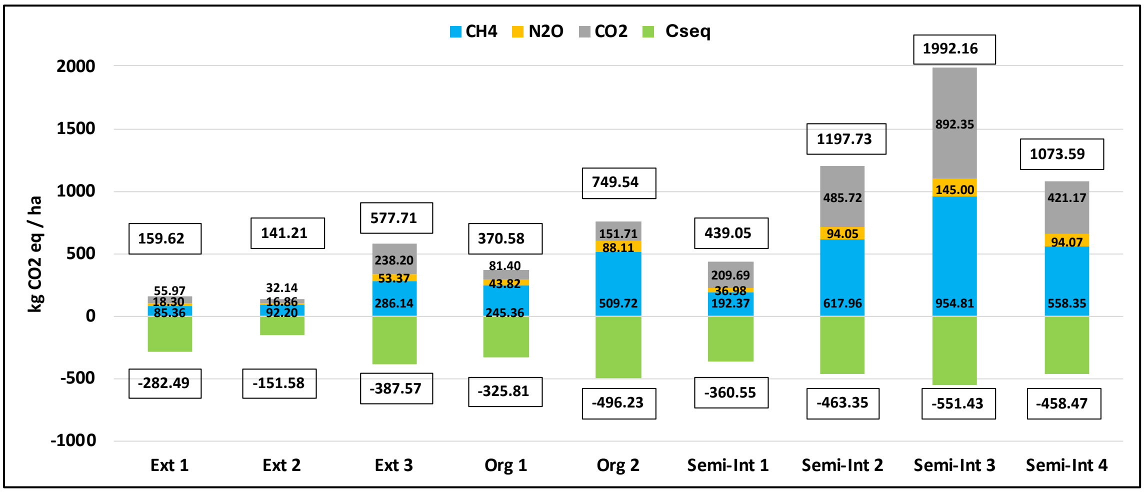

Figure S4. GHG Emissions Balance of the farms in the study in kg CO_2 eq/hectares and GWP proposed in the Fourth Assessment Report [3](AR4) (ha: hetares)

References

1. IPCC Climate Change 2014: Mitigation of Climate Change. Contribution of Working Group III to the Fifth Assessment Report of the Intergovernmental Panel on Climate Change [Edenhofer, O., R. Pichs-Madruga, Y. Sokona, E. Farahani, S. Kadner, K. Seyboth, A. Adler,; 2014;

2. IPCC Intergovernmental Panel on Climate Change (2022) P.R. Shukla, J. Skea, R. Slade, A. Al Khourdajie, R. van Diemen, D. McCollum, M. Pathak, S. Some, P. Vyas, R. Fradera, M. Belkacemi, A. Hasija, G. Lisboa, S. Luz, J. Malley (Eds.), Climate Change 2022: Miti; Intergovernmental Panel on Climate Change (IPCC), Ed.; Cambridge University Press, 2023;

3. IPCC Climate Change 2007: Mitigation. Contribution of Working Group III to the Fourth Assessment Report of the Inter- Governmental Panel on Climate Change [B. Metz, O.R. Davidson, P.R. Bosch, R. Dave, L.A. Meyer (Eds)], Cambridge University Press, Cambridge, U; 2007;.
